# Transient silencing of the *KASII* genes is feasible in *Nicotiana benthamiana* for metabolic engineering of wax ester composition

**DOI:** 10.1038/srep11213

**Published:** 2015-06-11

**Authors:** Selcuk Aslan, Per Hofvander, Paresh Dutta, Folke Sitbon, Chuanxin Sun

**Affiliations:** 1 Department of Plant Biology, Uppsala BioCenter, Linnean Centre for Plant Biology, Swedish University of Agricultural Sciences (SLU), Uppsala, Sweden; 2 Department of Plant Breeding, Swedish University of Agricultural Sciences (SLU), Alnarp, Sweden; 3 Department of Food Science, Uppsala BioCenter, Swedish University of Agricultural Sciences (SLU), Uppsala, Sweden

## Abstract

The beta-ketoacyl-ACP synthase II (KASII) is an enzyme in fatty acid biosynthesis, catalyzing the elongation of 16:0-acyl carrier protein (ACP) to 18:0-ACP in plastids. Mutations in *KASII* genes in higher plants can lead to lethality, which makes it difficult to utilize the gene for lipid metabolic engineering. We demonstrated previously that transient expression of plastid-directed fatty acyl reductases and wax ester synthases could result in different compositions of wax esters. We hypothesized that changing the ratio between C16 (palmitoyl-compounds) and C18 (stearoyl-compounds) in the plastidic acyl-ACP pool by inhibition of *KASII* expression would change the yield and composition of wax esters via substrate preference of the introduced enzymes. Here, we report that transient inhibition of *KASII* expression by three different RNAi constructs in leaves of *N. benthamiana* results in almost complete inhibition of *KASII* expression. The transient RNAi approach led to a shift of carbon flux from a pool of C18 fatty acids to C16, which significantly increased wax ester production in *AtFAR6*-containing combinations. The results demonstrate that transient inhibition of *KASII* in vegetative tissues of higher plants enables metabolic studies towards industrial production of lipids such as wax esters with specific quality and composition.

Oil crops are of great interest since they can provide a sustainable production of high-value oleochemicals such as wax esters and fatty alcohols for the chemical industry with similar hydrocarbon structures to those of conventional petrochemical products. Wax esters (WE) are highly hydrophobic neutral lipids that are composed of medium to very long chain alcohols esterified to a fatty acid moiety. They have excellent lubrication properties, oxidation stability, and high resistance to hydrolytic degradation. The unique physical properties of wax esters bring a high value to industrial applications within, for instance, the textile, cosmetic, packaging, ink, candle, drug, and food industries^1^,^2^,^3^. Wax esters are present in plants, animals, and microorganisms, where they serve a number of biological functions including prevention of water loss, protection against pathogens, insects, and UV radiation^4^,^5^, energy storage^6^, buoyancy density regulation^7^, and gland secretion in bird species^8^. Historically, the main source of wax esters for industrial applications was the spermaceti organ of sperm whales, until hunting of these animals was banned because the species came close to extinction. Nowadays, the industrial demand for wax esters from biological sources is mainly dependent on the carnauba palm (*Copernicia prunifera*) or the desert shrub jojoba (*Simmondsia chinensis*)^9^. The limited natural resources for plant wax esters, compared with the potential applications, provide high motivation to design and develop specialized wax esters for industrial purposes by metabolic engineering of additional plant species.

In plants, *de novo* fatty acid biosynthesis consists of a number of enzymatic reactions^10^,^11^ where C2-units (two carbon units) are added to a growing fatty acyl chain by a small β-ketoacyl-ACP synthase (KAS) family in plastids^12^,^13^. The reaction series commonly results in the formation of C16:0-ACP (palmitic) and C18:0-ACP (stearic) fatty acids. Three plastidial classes of KAS enzymes have been characterized. KASIII is responsible for condensation of C2:0-ACP to C4:0-ACP, KASI catalyzes reactions from C4:0-ACP up to C16:0-ACP, and finally KASII mediates the extension of C16:0-ACP to C18:0-ACP^13^. KAS families play a central role in determining the range of substrates for other enzymes^14^. Newly produced fatty acids are either released from plastids by the activity of acyl-ACP thioesterase (TE), known as FATA and FATB^10^, or further desaturated from C18:0-ACP to C18:1-ACP by stearoyl-ACP desaturase (Δ9 desaturase or SAD) activity^15^,^16^ (Fig. 1). The fatty acid 16:0-ACP is a substrate not only for KASII and FATB, but also for SAD^17^ and lysophosphatidyl acyltransferase (LPAAT)^18^. These enzymes together are important for determination of the fatty acid pool available to the lipid biosynthetic machinery in the cytosol. Research with the aim of characterizing KASII function and specificity has been performed in various species^12^,^13^,^14^,^15^, and has demonstrated that KASII has the highest affinity for C16:0 substrates^12^. In mesophyll cells of Arabidopsis, 69% of the C16:0-ACP pool was shown to be converted into C18:0-ACP^19^ by the function of KASII. Moreover, a partially deficient Arabidopsis mutant, *fab1*, which caused a reduction in KASII activity of 38.5%, led to an increase of 43.1% in leaf C16:0-ACP (palmitic aid) content^20^. However, complete inhibition of KASII activity in plants seems unattainable, as deficiency of KASII is lethal^12^,^15^.

Biosynthesis of wax esters generally comprises three distinctive stages. Precursors required for initiation of the process are synthesized via *de novo* fatty acid synthesis, so this part of wax ester biosynthesis is shared with other lipid biosynthetic pathways^4^,^5^. The second stage of the enzymatic process is conversion of an activated fatty acid to a fatty alcohol by the function of a fatty acyl reductase (FAR) enzyme. This reaction varies among organisms and can be performed by a single enzyme, or by two separate enzymatic reactions^3^,^21^,^22^,^23^,^24^. The final stage is the esterification by a wax synthase (WS) of fatty alcohols with fatty acids to form wax esters^23^,^25^. The composition of wax esters depends on the specific catalytic enzymes (FARs and WSs) and the availability of acyl-ACP substrates^26^. In an earlier study, we demonstrated the feasibility of producing different qualities and quantities of wax esters in chloroplast organelles of *Nicotiana benthamiana* leaves by a combination of several genes encoding enzymes for wax ester biosynthesis^27^. Arabidopsis FAR6 (AtFAR6) showed a high substrate preference for C16:0-ACP in the production of primary fatty alcohols, while *Marinobacter* FAR (MaFAR) was able to use both C16:0-ACP and C18:0-ACP at a similar rate^27^. In addition, PES2 (phytyl ester synthase2) in chloroplasts displayed high activity in esterification of primary alcohols and fatty acids for wax ester production in *N. benthamiana*.

Aiming at an increase in substrates for wax ester biosynthesis, we hypothesized that the pool of C16:0-ACP could be increased by transient inhibition of KASII activity. The increased C16:0-ACP pool could then be used by one of the alcohol-forming enzymes (AtFAR6 in particular, or MaFAR), thereby resulting in an elevated wax ester content. To investigate the role of KASII in production of wax ester quantity and quality in chloroplasts of *N. benthamiana*, we made three different RNAi constructs against *KASII* genes in *N. benthamiana* and infiltrated leaves to test whether the inhibition could lead to an increase in C16:0 levels. Our aim was to demonstrate that modulation of *KASII* expression is possible in plant vegetative tissues. This provides a rational experimental approach for increasing levels of C16:0 and decreasing levels of C18:0 in the metabolic engineering of wax ester synthesis for improvement of the specific composition and quality of wax esters for industrial purposes.

## Results

### Gene constructs and expression levels of *KASII* after transient RNAi silencing

Blast analysis at solgenomics (www.solgenomics.net) using DNA sequences of *Arabidopsis thaliana KASII* corresponding to GenBank accession AY081285 identified two putative *KASII* genes in the *N. benthamiana* genome. In order to test whether simultaneous inhibition of the two *KASII* genes was possible, three different RNAi constructs were made towards different regions of *KASII* using intron-spliced hairpin technology^28^. These constructs were denoted *KASIIRNAi-1*, -*2* and -*3* (Fig. 2a,b). They were infiltrated without or with our previous wax ester production system^27^ (including AtFAR6 + AtPES2 or MaFAR + AtPES2; Fig. 2c), where p19 + GFP infiltrated tissues were used as controls, and also co-infiltrated in each combination. Five days after infiltration, down-regulation of the two *KASII* genes (counted as *KASII* total) in the three *KASIIRNAi* experiments was assessed by quantitative real-time PCR (Fig. 3a). The results showed severe inhibition of *KASII* in each combination (Fig. 3a). Upon infiltration, the control leaves were affected, resulting in visible symptoms (slight bleaching in the infiltrated area and infiltration wound spots), but there was no difference compared with any additional *KASIIRNAi* gene constructs (Fig. 3b). These data show that the agro-infiltration strategy could be used for inhibition of *KASII* in studies of wax ester metabolic engineering in *N. benthamiana*.

### Increased wax ester levels by inhibition of *KASII*

We further investigated the consequences of *KASII* inhibition in our wax ester production system by examining total wax ester levels and compositions. Five days after infiltration, the production of wax esters was apparent after separation of total lipid extracts on thin layer chromatography (TLC) plates, while very low amounts of wax esters were observed in the controls (data not shown). The total amount of wax esters in the controls was small and infiltration of individual *KASII* RNAi constructs did not significantly change these levels (Fig. 4a, left panel).

In the experiments with *AtFAR6*-containing combinations, inhibition of *KASII* expression significantly increased wax ester production levels, but there was no significant increase for the combinations with *MaFAR*. The highest wax ester level in the combinations with *AtFAR6* was found for the combination *AtFAR6* + *PES2* + *RNAi-3*, with an amount of 1.62 nmol/mg per fresh weight (FW), corresponding to about 0.9% of leaf dry weight (DW) (Fig. 4a, middle panel). In general, all combinations with *MaFAR* produced a higher amount of wax esters compared with combinations with *AtFAR6* (Fig. 4a, right panel).

### Increased wax ester levels in relation to elevated C16/C18 ratio

The composition of wax esters was examined in order to monitor changes induced by *KASII* inhibition. We observed a significant increase in C16/C18 ratio in the small amount of wax esters in the control tissues where *KASIIRNAi* were expressed on their own without the wax ester synthesis capacity added (Fig. 4b, left panel).

Increased C16/C18 ratio was also observed when *AtFAR6* was utilized to supply fatty alcohols (Fig. 4, middle panel). The increase in wax ester production levels in these combinations was associated with increased C16/C18 ratio (Fig. 4b vs. Fig. 4a, middle panels). When *AtFAR6* was co-expressed in the combinations with *KASIIRNAi*, C16:0 fatty acids were increased by as much as 72% in the combination *AtFAR6* + *PES2* + *RNAi-3* (Supplementary Table S1) compared with the corresponding control (*AtFAR6* + *PES2*), and the total wax ester levels were increased by 73% in this specific combination (Fig. 4b, middle panel). *KASII* silencing did not significantly affect the C16/C18 ratio upon expression of *MaFAR* combinations (Fig. 4b, right panel).

### Triacylglycerol (TAG) and free alcohols after KASII inhibition

*De novo* fatty acid synthesis is a shared pathway in the production of other lipid molecules, and we expected that any release of fatty acids from the plastid could be used for TAG assembly. We thus further investigated TAG accumulation and composition in all gene combinations used in the transient expression system. While total TAG amounts were not changed significantly in any of the combinations (Fig. 4c), the C16/C18 ratio was significantly increased when *KASIIRNAi-3* was expressed with or without wax ester biosynthesis genes (Fig. 4d). However, this increase was not observed in the case of *KASIIRNAi-1* and *-2*.

The free fatty alcohols that were not utilized in wax ester synthesis in the infiltrated leaves were also analyzed in each gene combination and in the controls. In general, the total free alcohol level was not changed significantly by *KASII* inhibition, and was higher in *MaFAR*-infiltrated leaves than in *AtFAR6-*infiltrated leaves (Fig. 4e). The C16:0-OH/C18:0-OH ratio in control tissues was significantly changed upon infiltration of all three *KASIIRNAi* constructs (Fig. 4f, left panel), while the ratio was not significantly changed upon *KASII* inhibition in the combinations compared with the controls (Fig. 4f, middle and right panels). Free fatty alcohols were mainly composed of 16:0-OH and 18:0-OH species, as were the wax esters. The 16:0-OH species dominated in the *AtFAR6*-infiltrated leaves, while equal amounts of C16:0-OH and C18:0-OH were observed in the *MaFAR*-infiltrated combinations (Supplementary Table S1).

### Increased C16/C18 ratio in total lipid extracts of leaf tissue after *KASII* inhibition

To further investigate the effects of *KASII* inhibition on the fatty acid composition of total lipids related to KASII activity (*i.e.* C16/C18 ratio) in leaf tissue, we examined fatty acid composition in total leaf lipid extracts. Interestingly, the C16/C18 ratio in the total lipid samples was significantly increased after *KASII* inhibition, regardless of presence of *AtFAR6*, *MaFAR*, or *PES2* (Fig. 5). Furthermore, a higher C16/C18 ratio was observed in samples when additional wax ester biosynthesis genes were included. The change in the *KASIIRNAi-3* sample without addition of *AtFAR6*, *MaFAR* or *PES2* further demonstrates the role of *KASII* in determination of fatty acid composition of other lipids in total lipids as the lipid compounds examined, *i.e.*, wax esters and free fatty alcohols were present in trace amounts in the *KASIIRNAi-3* sample (Fig. 4).

## Discussion

Transient gene expression systems can be used to rapidly assess simultaneous effects of several genes on the quality and quantity of a specific product. The results presented here provide evidence that hairpin-RNAi targeting the *KASII* genes in *Nicotiana benthamiana* leaf tissues makes more palmitic acid available for metabolism of complex products and, as a consequence, elevates the C16/C18 ratio in wax esters. These results provide new insights into the use of transient approaches to manipulate fatty acid synthesis for both quality and quantity, with emphasis on enhancing production of oleochemicals in leaves of *N. benthamiana*. Complete inhibition of the *KASII* genes can lead to lethality in stable transformation systems. It has been shown that in Arabidopsis transformants, strong seed-specific hairpin-RNAi reduction of *FAB1* (alias *KASII*) expression is lethal, while lines with less severe reduction of *FAB1* expression show normal embryo development and increased palmitic acid levels^15^. We showed here that transient expression of any *KASIIRNAi* gene did not affect leaf health differently from the control five days after infiltration (Fig. 3b). To avoid detrimental effects on plant vigor in future applications using *KASII* inhibition in stable transformation, we suggest that genotypes with an appropriate degree of silencing be selected among transformants or that a developmentally regulated or inducible promoter be used. Inhibition efficiency of *KASIIRNAi* was apparently higher when co-expressed with additional genes *FAR* and *PES* (Fig. 3a). P19 has been found to form a homo-dimer which binds siRNAs^29^,^30^. According to Fig. 2C of Naim *et al.*^31^, co-infiltration with p19 enhances expression of GFP, but it also makes hpGFP inhibition of GFP less efficient. Therefore a possible explanation could be that when *KASIIRNAi* is expressed on its own together with p19, inhibition is less efficient than if additional products such as *FAR* and *PES* are expressed. There could thus be a titration effect towards p19.

**Figure 1:**
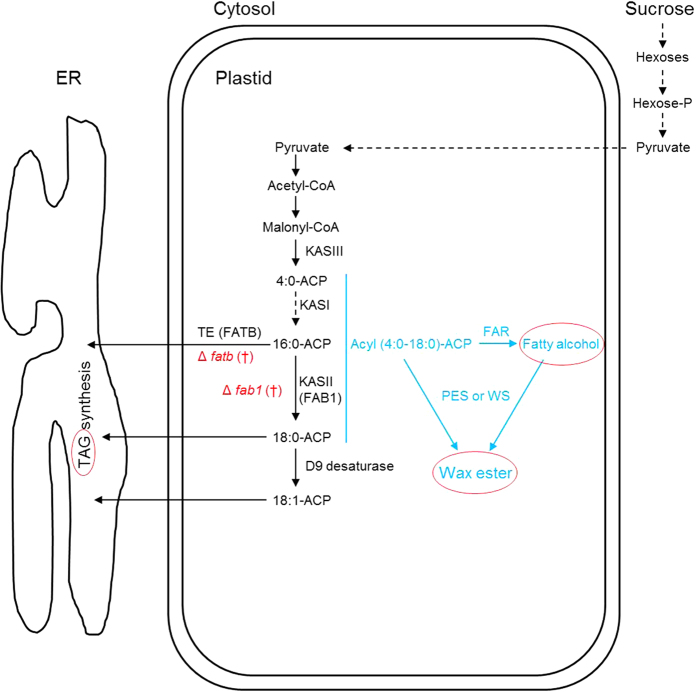
Schematic overview of manipulation of enzymatic steps and estimated co-integration of fatty acids into different metabolites through *de novo* fatty acid synthesis in a plastid. Mutations with a lethal phenotype are shown in red. Metabolites monitored in the present study are shown in red circles.

From this study we conclude that composition and quality of wax esters can be modified by transient *KASIIRNAi* inhibition. Through metabolic engineering, the fatty acid composition of total lipids other than wax esters can also be altered via this approach. Moreover, quantity of wax esters can be improved by using certain enzymes (*e.g.*, AtFAR6) that prefer palmitic components. To our knowledge, the present work is the first report on metabolic engineering of wax ester compositions by transient silencing of the *KASII* genes. In future development work to ensure higher substrate supply and further enhance production of wax esters, it would be of great interest to study inhibition of acyl-ACP thioesterases^10^,^15^,^16^,^32^ and/or stearoyl-ACP (SAD)^15^,^16^,^17^,^33^ desaturase activity in our wax ester production system^27^ (see also Fig. 1).

## Methods

### Molecular cloning of genes

**Figure 2:**
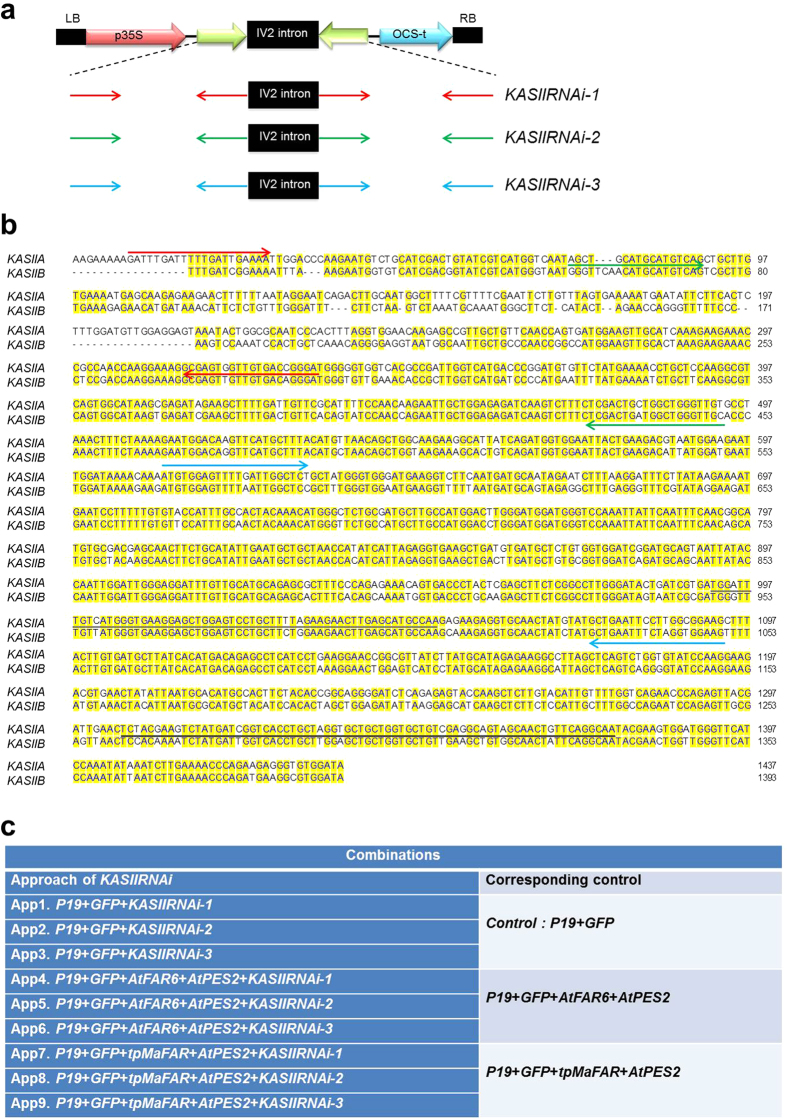
Constructions of *KASIIRNAi-1, KASIIRNAi -2, and KASIIRNAi -3* and experimental design of agro-infiltration in *Nicotiana benthamiana* leaves. (**a**) Schematic drawing of the three *KASIIRNAi* constructs used to create an hairpin RNAi. The sequences of sense and antisense regions of individual constructs were inserted on opposite sites flanking the IV2 intron in the expression vector *pGEMIV23Z*^41^. The RNAi construct thus created was then inserted into Ti-plasmid pART27. OCS-t: Octopine synthase terminator; LB and RB: left and right T-DNA border, respectively. (**b**) Gene sequences of *KASIIA* and *KASIIB* in *N. benthamiana* genome. The areas (primers) for construction are highlighted in red for *KASIIRNAi-1*, green for *KASIIRNAi-2*, and blue for *KASIIRNAi-3*. Black and grey bars indicate the area of gene sequences used for detection of transcript levels of *KASII* gene in *N. benthamiana* by quantitative PCR. (**c**) Gene constructs and different combinations used for infiltration and transient expression in *N. benthamiana* leaf tissues.

**Figure 3:**
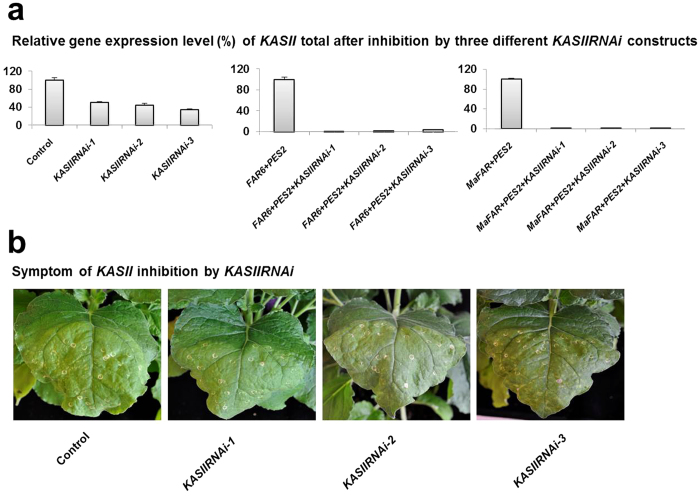
Transcript levels and appearance of plant tissues of *N. benthamiana* after agro-infiltration. (**a**) Quantitative PCR analysis of individual genes used in each combination and controls in *N. benthamiana* leaf tissue. Individual gene expression levels were normalized to the expression of *Actin* (*ACT*) gene. Relative gene expression level shown as %. Relative mean values from three independent biological experiments, analyzed in technical triplicates. (**b**) Photos of leaf tissues of *N. benthamiana* five days post infiltration. The corresponding control is described in Fig. 2c. Similar symptoms (bleaching in the infiltrated area with infiltration wound spots) were observed in control leaves and leaves with additional *KASIIRNAi* gene constructs.

**Figure 4:**
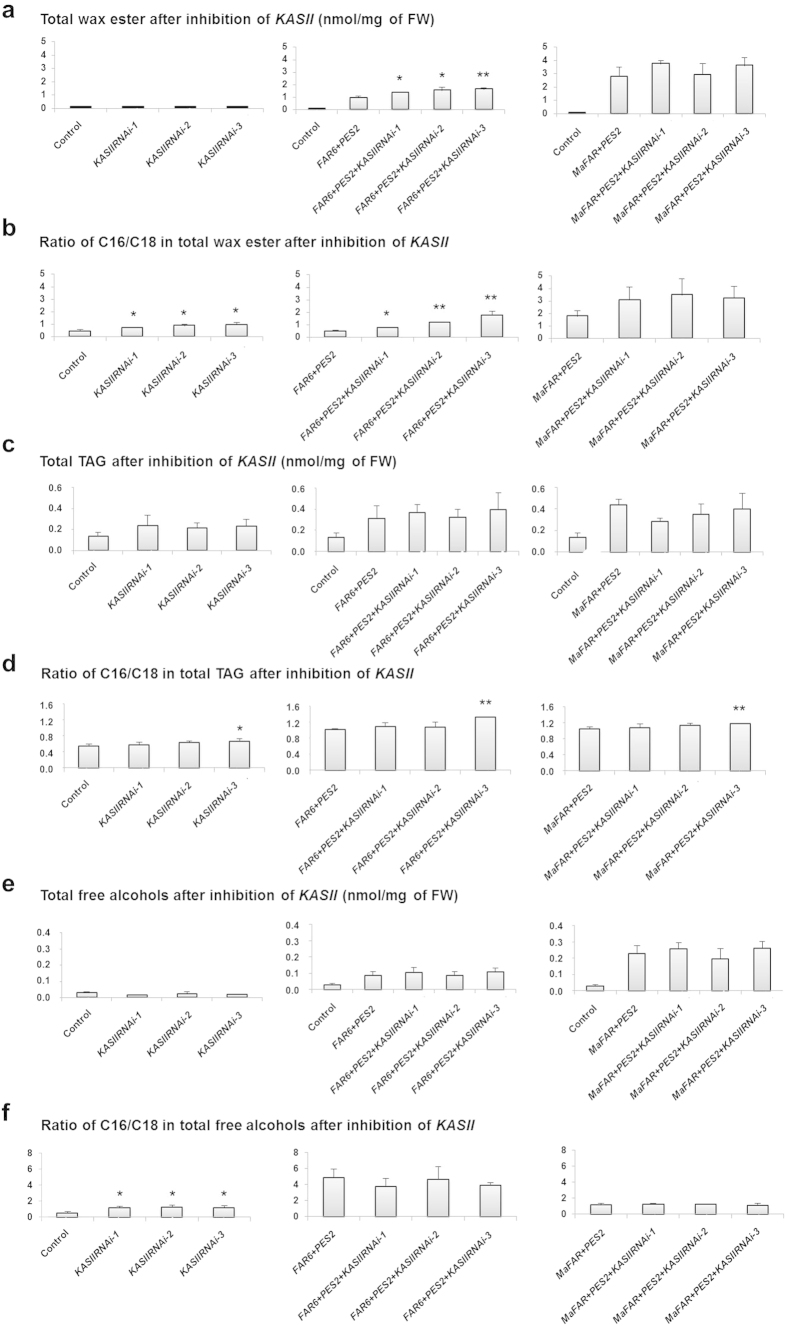
Metabolite levels after *KASIIRNAi inhibition in N. benthamiana* leaves. (**a**) Total wax ester content in leaves. (**b**) Total C16/C18 fatty acid ratio in wax esters. (**c**) Total triacylglycerol (TAG) production in different combinations. (**d**) Total C16/C18 fatty acid ratio in TAGs. (**e**) Total free alcohol found in *Nicotiana benthamiana* leaf extracts. (**f**) Total C16/C18 fatty alcohol ratio in free alcohols. All data presented are mean (with SD) of biological triplicates. Statistical analyses for each RNAi sample were performed against the corresponding control. Only statistically significant increases are indicated, (* P < 0.05, **P < 0.01). The corresponding control is described in Fig. 2c.

**Figure 5:**
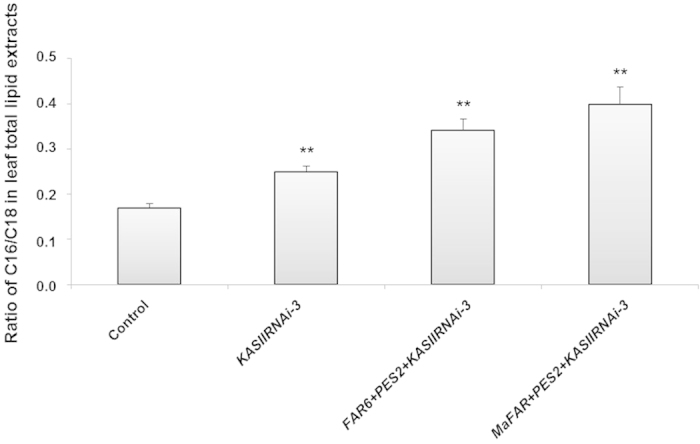
Fatty acid composition of total lipids from leaf tissues. The C16/C18 fatty acid ratio in total lipids related to KASII activities is shown. All data presented are mean (with SD) of biological triplicates. Statistical analyses for each RNAi sample were performed against the control. Statistically significant increases are indicated (**P < 0.01). The control is described in Fig. 2c.

The construction procedure of binary vectors containing the genes in wax ester biosynthesis, *AtFAR6*, *MaFAR*, and *AtPES2* driven by the constitutive CaMV-35 S promoter, was performed as described previously^27^. The constructs containing the tomato bushy stunt virus p19 viral silencing suppressor and GFP^34^ genes under control of the CaMV-35S promoter were kindly provided by Dr. Craig C. Wood (CSIRO, Australia). In order to get the sequences of β-ketoacyl-ACP synthase II genes in the *Nicotiana benthamiana* genome, DNA sequences of *Arabidopsis thaliana* corresponding to GenBank accession AY081285 were blasted on http://solgenomics.net against *N. benthamiana* genome. The blasting results revealed two genes in *N. benthamiana* genome with 81% identity (GenBank ID#NbS00033926g0004.1), and 80% identity (GenBank ID#NbS00001834g0011.1), respectively, to Arabidopsis *KASII*. The RNAi constructs against different coding regions (denoted *KASIIRNAi-1, KASIIRNAi-2*, and *KASIIRNAi-3*) were cloned as inverted repeat hairpin constructs. The DNA sequences of sense and antisense regions (325 bp for *KASIIRNAi-1,* 396 bp for *KASIIRNAi-2*, and 483 bp for *KASIIRNAi-3*) were amplified by PCR using the wild-type cDNA of *N. benthamiana* as a template. The primers used for cloning of individual constructs are listed in Supplementary Table S2. The amplified PCR fragments were sub-cloned into chemically competent *E. coli* TOP10 cells using the TOPO TA Cloning Kit (Invitrogen, Carlsbad, CA, USA). The expression cassette in the binary vector was digested with *BamHI* and *XbaI* for the sense region and *SacI* and *KpnI* for the antisense regions, and then inserted between the corresponding sites of expression vector *pGEMIV23Z*, which includes the IV2 intron^35^ sequences. The final construct was digested with *XbaI* and *SalI* (Fermentas), and then inserted into the corresponding sites of expression vector pART27^36^, resulting in plasmids *p35S-KASIIRNAi-1*, *p35S-KASIIRNAi-2*, and *p35S-KASIIRNAi-3* (Fig. 2). Final constructs were verified by DNA sequencing (Macrogen Europe, Amsterdam, the Netherlands). All constructs used in this study were transformed into *Agrobacterium tumefaciens* strain GV3101 using the freeze-thaw method.

### Plant materials and transient gene expression in *N. benthamiana* leaves

*Nicotiana benthamiana* plants were grown at 25 °C in a 16 h light:8 h dark regime in a phytotron. Light intensity was 320 μmol/m^2^/s and relative humidity (RH) was 60%. The plants were grown in 21 × 16 cm pots and watered regularly with 1% of NPK macronutrients^37^. The second-from-top leaves of 6-week-old plants were used for agro-infiltration.

Transient expression of genes in *N. benthamiana* leaves by the agro-infiltration method was performed as described previously^27^,^34^,^38^ with small modifications. *Agrobacterium tumefaciens* cultures containing the gene(s) of interest were mixed so that the final OD_600_ of each culture was equal to 0.2 prior to infiltration. The different combinations of the genes and controls are listed in Fig. 2c. In every combination, a p19 construct for silencing effect and GFP construct for identifying the infiltrated areas were included. After infiltration, *N. benthamiana* plants were grown for a further five days^39^,^40^, infiltrated areas were exposed to UV light, and GFP-expressing regions were excised, freeze-dried, weighed, and stored at −80 °C for further analyses.

### Fatty alcohol and lipid analysis by TLC and gas chromatography (GC)

The leaf areas that showed fluorescence under UV light were excised, homogenized in 3.75 ml methanol/chloroform (2:1 v/v) and 1 ml 0.15 M acetic acid, and mixed with chloroform for extraction as described^41^. Total lipid extracts corresponding to 100 mg fresh weight of leaves were applied to TLC (silica 60, Merck) separation. Free fatty alcohols were separated with hexane:diethylether:acetic acid (85:15:1, v/v/v) as mobile phase. The alcohol spots were located on the plates by co-migration with the heptadecanol (C17:0-OH) standard sprayed with water. Free alcohols were eluted from the silica material with methanol and extracted with chloroform as described previously^27^. Total lipid extracts corresponding to 20 mg fresh weight of leaves were used for analysis of total fatty acid composition. The leaf extracts were dried under nitrogen flow, dissolved in 500 μl n-hexane, methylated, and analyzed by GC as described below. The extracted fatty alcohols, wax esters, TAGs, and fatty acids in total leaf lipids were analyzed by GC as described previously^27^. As internal standard, added prior to lipid extraction, 5 nmol of heptadecanoyl heptadecanoate was used for wax esters, 5 nmol of heptadecanol for free alcohols, and 5 nmol of triheptadecanoin for TAGs and total leaf fatty acids.

### RNA isolation and gene expression analysis

Total RNA from the collected leaf materials was extracted by grinding approximately 100 mg of fresh leaf tissue in liquid nitrogen and applying the Spectrum™ Plant Total RNA Kit (Sigma-Aldrich, St. Louis, MO, US). All samples were treated with DNase I (Sigma-Aldrich, St. Louis, MO, US) to remove any residues of DNA contamination. The first-strand cDNA was performed using the qScript cDNA Synthesis kit following the manufacturer’s instructions (Quanta Biosciences, Gaithersburg, MD, USA), and 1 μg of total RNA was used as a template in 20 μl reaction volume. Quantitative RT-PCR (qRT-PCR) reactions (20 μl) included SYBR Green PCR master mix (Applied Biosystems, Life Technologies Europe BV, Stockholm, Sweden), supplemented with 5 μM primer (Supplementary Table S2) and 1 μl cDNA as template, which was diluted up to 200 μl after synthesis.

Primers for qRT-PCR were designed using the tool of Roche Applied Sciences. qRT-PCR primers were analyzed by blasting against the *N. benthamiana* genome sequences on the solgenomics network website (http://solgenomics.net/). Reaction mixtures without cDNA were used as a negative control. PCR was performed as follows: 10 min at 50 °C, 5 min at 95 °C, 40 cycles of 10 s at 95 °C and 30 s at 60 °C, and finally 1 min at 95 °C, in 96-well optical reaction plates (Applied Biosystems, Life Technologies Europe BV, Stockholm, Sweden). The specificity of the reactions and the amplicon identities were verified by melting curve analysis. The data were analyzed by the comparative C_T_ method^42^ with PCR efficiency correction, which was determined based on the slope of standard curves. The gene expression level by qRT-PCR was normalized using the gene *Actin* (*ACT*) gene^43^ and in some cases the fold-differences in the transcript levels, and mean standard error was calculated as described previously^42^. Primer combinations and reactions were shown to be specific for the purposes of the experiment and therefore no signal was found in the control tissues.

### Statistical analysis

One-way ANOVA was used for statistical analysis.

## Additional Information

**How to cite this article**: Aslan, S. *et al.* Transient silencing of the *KASII* genes is feasible in *Nicotiana benthamiana* for metabolic engineering of wax ester composition. *Sci. Rep.* **5**, 11213; doi: 10.1038/srep11213 (2015).

## Supplementary Material

Supplementary Information

Supplementary Information
