# Supplementary material for: Transient silencing of the KASII genes is feasible in Nicotiana benthamiana for metabolic engineering of wax ester composition
Source: Sci Rep. 2015 Jun 11;5:11213. doi: 10.1038/srep11213 (PMC4463015; doi:10.1038/srep11213)
Supplement: Supplementary Information [file srep11213-s1.pdf]

Supplementary information

Transient silencing of the *KASII* genes is feasible in *Nicotiana benthamiana* for metabolic engineering of wax ester composition

Selcuk Aslan[1], Per Hofvander[2], Paresh Dutta[3], Folke Sitbon[1], Chuanxin Sun[1*]

[1]Department of Plant Biology, Uppsala BioCenter, Linnean Centre for Plant Biology, Swedish University of Agricultural Sciences (SLU), Uppsala, Sweden

[2]Department of Plant Breeding, Swedish University of Agricultural Sciences (SLU), Alnarp, Sweden

[3]Department of Food Science, Uppsala BioCenter, Swedish University of Agricultural Sciences (SLU), Uppsala, Sweden

*Correspondence

Email addresses:

chuanxin.sun@slu.se

Table S1. Palmitoyl (C16) and stearoyl (C18) compounds in wax esters, triacylglycerols. and free fatty alcohols in different combinations after agroinfiltration in *Nicotiana benthamiana* leaves. Note: The corresponding control is described as in Figure 2c.

	Wax esters (nmol/mg of FW)					
Combinations	16.0	18.0	18.1	18.2	18.3	Total 18
Control	0.015 ± 0.002	0.011 ± 0.006	0.003 ± 0.001	0.017 ± 0.002	0.008 ± 0.002	0.038 ± 0.011
KASIIRNAi-1	0.036 ± 0.002	0.011 ± 0.001	0.002 ± 0.001	0.002 ± 0.001	0.035 ± 0.004	0.050 ± 0.007
KASIIRNAi-2	0.036 ± 0.006	0.009 ± 0.003	0.004 ± 0.003	0.002 ± 0.001	0.025 ± 0.010	0.040 ± 0.016
KASIIRNAi-3	0.034 ± 0.003	0.006 ± 0.000	0.002 ± 0.000	0.002 ± 0.000	0.020 ± 0.017	0.030 ± 0.018
AtFAR6+AtPES2	0.073 ± 0.012	0.012 ± 0.005	0.002 ± 0.000	0.063 ± 0.021	0.067 ± 0.016	0.153 ± 0.042
AtFAR6+AtPES2+KASIIRNAi-1	0.108 ± 0.013	0.027 ± 0.005	0.004 ± 0.001	0.055 ± 0.007	0.057 ± 0.002	0.143 ± 0.015
AtFAR6+AtPES2+KASIIRNAi-2	0.115 ± 0.020	0.027 ± 0.003	0.006 ± 0.002	0.037 ± 0.012	0.027 ± 0.003	0.097 ± 0.020
AtFAR6+AtPES2+KASIIRNAi-3	0.126 ± 0.013	0.013 ± 0.007	0.004 ± 0.001	0.022 ± 0.001	0.034 ± 0.005	0.073 ± 0.014
tpMaFAR+AtPES2	0.224 ± 0.064	0.024 ± 0.004	0.004 ± 0.000	0.043 ± 0.002	0.054 ± 0.003	0.125 ± 0.009
tpMaFAR+AtPES2+KASIIRNAi-1	0.257 ± 0.015	0.026 ± 0.002	0.004 ± 0.001	0.022 ± 0.013	0.037 ± 0.015	0.090 ± 0.031
tpMaFAR+AtPES2+KASIIRNAi-2	0.210 ± 0.038	0.040 ± 0.027	0.006 ± 0.001	0.008 ± 0.003	0.015 ± 0.007	0.070 ± 0.038
tpMaFAR+AtPES2+KASIIRNAi-3	0.260 ± 0.029	0.026 ± 0.005	0.005 ± 0.001	0.024 ± 0.007	0.030 ± 0.016	0.086 ± 0.029

Table S1. Continued.

Triacylglycerols (nmol/mg FW)						
Combinations	16.0	18.0	18.1	18.2	18.3	Total 18
Control	0.041 ± 0.010	0.009 ± 0.001	0.002 ± 0.001	0.019 ± 0.008	0.045 ± 0.014	0.075 ± 0.024
KASIIRNAi-1	0.083 ± 0.031	0.014 ± 0.003	0.001 ± 0.000	0.038 ± 0.019	0.095 ± 0.044	0.148 ± 0.067
KASIIRNAi-2	0.081 ± 0.014	0.016 ± 0.007	0.001 ± 0.001	0.030 ± 0.006	0.080 ± 0.016	0.127 ± 0.030
KASIIRNAi-3	0.089 ± 0.022	0.014 ± 0.004	0.001 ± 0.000	0.030 ± 0.009	0.089 ± 0.028	0.134 ± 0.042
AtFAR6+AtPES2	0.146 ± 0.052	0.024 ± 0.009	0.001 ± 0.000	0.033 ± 0.013	0.088 ± 0.036	0.146 ± 0.058
AtFAR6+AtPES2+KASIIRNAi-1	0.179 ± 0.041	0.024 ± 0.007	0.001 ± 0.000	0.033 ± 0.007	0.105 ± 0.014	0.163 ± 0.027
AtFAR6+AtPES2+KASIIRNAi-2	0.156 ± 0.042	0.017 ± 0.005	0.001 ± 0.000	0.028 ± 0.010	0.097 ± 0.015	0.143 ± 0.031
AtFAR6+AtPES2+KASIIRNAi-3	0.208 ± 0.084	0.019 ± 0.007	0.001 ± 0.000	0.030 ± 0.012	0.106 ± 0.043	0.156 ± 0.062
tpMaFAR+AtPES2	0.210 ± 0.017	0.035 ± 0.001	0.002 ± 0.001	0.036 ± 0.004	0.125 ± 0.019	0.198 ± 0.026
tpMaFAR+AtPES2+KASIIRNAi-1	0.130 ± 0.018	0.021 ± 0.004	0.001 ± 0.000	0.020 ± 0.002	0.078 ± 0.007	0.119 ± 0.013
tpMaFAR+AtPES2+KASIIRNAi-2	0.173 ± 0.040	0.024 ± 0.007	0.002 ± 0.001	0.025 ± 0.006	0.101 ± 0.027	0.152 ± 0.041
tpMaFAR+AtPES2+KASIIRNAi-3	0.197 ± 0.070	0.025 ± 0.009	0.002 ± 0.001	0.028 ± 0.010	0.111 ± 0.040	0.166 ± 0.060

Free fatty alcohols (nmol/mg FW)		
Combinations	16:0-OH	18:0-OH
Control	0.004 ± 0.002	0.008 ± 0.001
KASIIRNAi-1	0.002 ± 0.000	0.002 ± 0.000
KASIIRNAi-2	0.002 ± 0.001	0.002 ± 0.000
KASIIRNAi-3	0.002 ± 0.000	0.002 ± 0.000
AtFAR6+AtPES2	0.055 ± 0.013	0.011 ± 0.001
AtFAR6+AtPES2+KASIIRNAi-1	0.062 ± 0.019	0.016 ± 0.002
AtFAR6+AtPES2+KASIIRNAi-2	0.054 ± 0.016	0.012 ± 0.002
AtFAR6+AtPES2+KASIIRNAi-3	0.060 ± 0.005	0.015 ± 0.001
tpMaFAR+AtPES2	0.099 ± 0.017	0.083 ± 0.011
tpMaFAR+AtPES2+KASIIRNAi-1	0.122 ± 0.017	0.096 ± 0.009
tpMaFAR+AtPES2+KASIIRNAi-2	0.089 ± 0.028	0.073 ± 0.026
tpMaFAR+AtPES2+KASIIRNAi-3	0.114 ± 0.025	0.104 ± 0.013

Table S2. Oligonucleotides used in this study.

Primers used for construction of *KASIIRNAi*.			
Construct	Primer	Sequence 5´→3´	
KASIIRNAi-1	Forward	AAATCTAGAGATTTGATTTTTGATTGAAA	
	Reverse	AAAGGATCCTCCCGGTCACAACCACTCGC	
KASIIRNAi-1	Forward	AAAGAGCTCCTCGAGGATTTGATTTTTGATTGAAA	
	Reverse	AAAGGTACCTCCCGGTCACAACCACTCGC	
KASIIRNAi-2	Forward	AAA TCTAGAGGGTTCAACATGCATGTCAG	
	Reverse	AAAGGATCCGCAACCCAGCCATCAGTCGA	
KASIIRNAi-2	Forward	AAAGAGCTCCTCGAGGGGTTCAACATGCATGTCAG	
	Reverse	AAAGGTACCGCAACCCAGCCATCAGTCGA	
KASIIRNAi-3	Forward	AAATCTAGAATGTGGAGTTTTAATTGGCT	
	Reverse	AAAGGATCCATTCAAACTAGAAATTCAGC	
KASIIRNAi-3	Forward	AAAGAGCTCGTCGACATGTGGAGTTTTAATTGGCT	
	Reverse	TTTGGTACCCTTCGATCTAGAAATTCAGC	
Primers used for qRT-PCR analysis			
Gene	Primer	Sequence 5´→3´	Reference/Acc. No.
Actin	Forward	TCCTGATGGGCAAGTGATTAC	Liu and Shi et al. 2012[49]
	Reverse	TTGTATGTGGTCTCGTGGATTC	
FAR6	Forward	TCACTTCTGCTTTATCGAACACA	At3g56700
	Reverse	CTCTTTCCCTTCGTGCTCAA	
tpMaFAR	Forward	CCTGACGGATCGTGTTCTG	YP_959486
	Reverse	GACTGCGTGGTATCCAGGTT	

Table S2. Continued.

PES2	Forward	TGGAAAAGGCAACAAAGGAC	At3g26840
	Reverse	TTTCCCAATTCGCTCTCATC	
KASII after *KASIIRNAi-1* inhibition	Forward	TGGATTTGTCATGGGTGAAG	This work
	Reverse	TTGGCATGCTCAAGTTCTTCT	This work
KASII after *KASIIRNAi-2/3* inhibition	Forward	TCCACAAAATCTATGATTGGTC A	This work
